# Identification of key clinical features for pediatric respiratory syncytial virus infection using machine learning

**DOI:** 10.1186/s12887-026-06659-z

**Published:** 2026-02-27

**Authors:** Yoshifumi Miyagi, Yuichi Morimoto, Eiichiro Satake, Satoru Iwashima, Yasuyuki Yano, Ryosuke Urabe, Atsushi Kitagawa, Hiroyuki Kato, Kentoku Kin

**Affiliations:** 1 Department of Pediatrics, Haibara General Hospital, Makinohara City, , Shizuoka Prefecture 2887-1 Hosoe Japan; 2 Department of Pediatrics, Faculty of Medicine, Kindai University, Higashiosaka, Osaka Japan; 3 Genetics and Epidemiology, Joslin Diabetes Center, Boston, MA USA; 4 Department of Medicine, Harvard Medical School, Boston, MA USA; 5 Department of Pediatrics, Chutoen General Medical Center, Kakegawa, Shizuoka Japan

**Keywords:** Respiratory syncytial virus, Machine learning, Pediatric patients

## Abstract

**Background:**

Respiratory syncytial virus (RSV) is the leading cause of lower respiratory infections in children. However, current diagnosis currently relies heavily on the clinicians’ subjective judgment, potentially delaying appropriate intervention. Identifying the primary clinical features of RSV in hospitalized pediatric patients can aid triage. Therefore, we analyzed a publicly available dataset comprising 768 pediatric patients, 135 of whom had confirmed RSV infection.

**Methods:**

A binary classification model was developed using the CatBoostClassifier algorithm and evaluated through five-fold cross-validation. Feature importance was assessed using SHapley Additive exPlanations (SHAP) values, and the threshold values for the top features were derived from the dependency plots.

**Results:**

The model achieved an average area under the receiver operating characteristic curve (AUC) of 0.770, indicating a moderate predictive performance. The three most important features are weight, respiratory rate, and oxygen saturation (SpO₂). A simplified scoring system based on thresholds for weight (≤ 10 kg), respiratory rate (≥ 50 breaths/min), and SpO₂ (≤ 97%) achieved an AUC of 0.726.

**Conclusions:**

These findings suggest that machine learning can identify clinically meaningful features for RSV prediction in hospitalized children with severe pneumonia. The proposed scoring system may complement conventional, existing scoring approaches. However, prospective validation is required to confirm its generalizability and clinical utility.

**Supplementary Information:**

The online version contains supplementary material available at 10.1186/s12887-026-06659-z.

## Background

Respiratory syncytial virus (RSV) is a common respiratory pathogen responsible for more than 30 million lower respiratory tract infections and approximately 3 million hospitalizations worldwide each year [1]. It is the leading cause of lower respiratory tract infections in children younger than five years of age and one of the primary causes of mortality in infants younger than under one year [2]. The monoclonal antibody, palivizumab, has been approved by the U.S. Food and Drug Administration for the prevention of severe RSV infections in specific high-risk pediatric populations. However, its use is not indicated for children older than two years of age or adults [3]. Given the vulnerability of affected populations, a clear understanding of the clinical presentation of RSV is essential for identifying individuals at risk of developing severe disease.

The most commonly reported signs and symptoms of RSV infection in children include nasal discharge or congestion, cough, fever, and feeding difficulties [2]. In infants, one study observed cough in 96% of cases and wheezing in 52% [4], while other studies reported much lower frequencies, with cough in only 3–4% [5] and wheezing in just 6% [6]. Few studies have focused on children aged 3–5 years. However, a study conducted in Kenya found fever and crackles in more than 40% of children younger than five years of age [7]. Although several reviews have attempted to identify common symptom patterns, assessing trends in clinical features remains difficult because of variability in symptoms and the limited ability of young children to articulate their experiences [2].

The use of rapid diagnostic tests can reduce costs and expedite clinical decision-making when appropriately applied [8]. However, their relatively low sensitivities remain a concern [9, 10]. For example, Chartrand et al. reported that the sensitivity of RSV antigen tests in children varied according to the test kit used, with a pooled sensitivity of 81% (95% CI: 78–84%) [9]. Therefore, a comprehensive clinical risk assessment remains essential for accurate diagnosis. Creating a scoring system could offer a solution. In particular, in settings where it is difficult to perform rapid antigen testing or polymerase chain reaction (PCR) testing for all patients, making it essential to prioritize tests for patients with high scores. This may facilitate efficient allocation of testing resources and early isolation. Additionally, in outpatient and emergency settings, the system facilitates rapid decisions-making regarding oxygen administration and admission. This allows its introduction as a triage support tool.

Despite the broad range of symptoms associated with RSV infections and their overlap with those of other viral infections, no standardized clinical indicators or scoring systems currently exist to guide suspicion or diagnosis with sufficient accuracy. Furthermore, the pathophysiology, airway anatomy, immune maturation, and symptom presentation in infants aged younger than 12 months differ significantly from those of older children. In routine pediatric practice, diagnosis often depends on the clinician’s subjective judgment, which may delay timely interventions. Importantly, while most RSV infections are mild and manageable on an outpatient basis, some children experience rapid deterioration requiring hospitalization, oxygen therapy, or intensive monitoring. This study was not designed to diagnose all cases of RSV infection including those in outpatients, but rather to support triage.

To address this issue, we applied machine learning (ML) techniques to identify key clinical features associated with RSV infection in children.

## Methods

### Data retrieval

A publicly available dataset of pediatric respiratory infections was obtained from Mendeley Data (https://data.mendeley.com/) [11]. We initially analyzed data from 801 pediatric patients. The study participants were children hospitalized at Rabat Children’s Hospital in Morocco for treatment following a diagnosis of severe pneumonia according to the World Health Organization (WHO) criteria. After excluding patients with chronic underlying conditions, 768 participants, including 135 with RSV infections, were included in the final analysis. RSV infection diagnosis was performed using the TrueScience™ RespiFinderR 19 Pathogen Identification Panel (CATALOGUE N° 4,460,382). This is a multiplex PCR/multiplex ligation-dependent probe amplification (MLPA)-based panel diagnostic kit targeting viruses and certain bacteria. The dataset contains cases of respiratory infections caused by RSV, rhinovirus, adenovirus, coronavirus, influenza virus, parainfluenza virus, and human metapneumovirus. The 135 RSV-positive cases, including coinfections, were designated as the case group, whilethe remaining 633 patients without RSV infection were considered controls. Of the 135 RSV cases, 84 involved RSV infection alone and 51 involved coinfection with other viruses.

The following 20 clinical and demographic features were used for analysis:

‘Age (months),’ ‘Gender,’ ‘Known asthmatic patient,’ ‘History of vomiting,’ ‘History of diarrhea,’ ‘History of cough,’ ‘History of rhinorrhea,’ ‘Weight (kg),’ ‘Height (cm),’ ‘Oxygen saturation (SpO₂) at admission,’ ‘Axillary temperature (°C),’ ‘Respiratory rate,’ ‘Heart rate,’ ‘Laryngeal stridor,’ ‘Rhonchi,’ ‘Crackles,’ ‘Wheezing,’ ‘Signs of dehydration,’ ‘Cyanosis,’ and ‘Nasal flaring’. To assess multicollinearity among continuous variables, we calculated the variance inflation factor (VIF). Ethical approval was not required, as only publicly available anonymized data were used (Mendeley Data repository).

### Machine learning and feature selection

We performed binary classification using the CatBoostClassifier with default settings and a fixed random seed of 42 [12]. For missing values, the CatBoostClassifier learns the optimal splitting direction based on gradient statistics for numerical features. It treated categorical variables as independent categories converted using target encoding techniques [12]. Categorical features were specified using the “cat_features” parameter. To address class imbalance in the dataset, we applied a stratified five-fold cross-validation. To minimize confounding from other viral infections, we conducted a comparative analysis of RSV-only cases (*n* = 84) with RSV-negative controls (*n* = 633). The feature importance was assessed using SHapley Additive exPlanations (SHAP) values averaged across five folds [13]. We identified three features that consistently demonstrated high importance across all the folds as robust predictors. The area under the receiver operating characteristic (ROC) curve (AUC) was used to evaluate the model performance, and the average AUC across folds was reported.

### Calibration plot

To evaluate the reliability of the predicted probabilities, we performed probability calibration using the CalibratedClassifierCV class in the scikit-learn software. The base classifier was trained using five-fold cross-validation, and isotonic regression (method = 'isotonic') was used for calibration. The calibrator was further cross-validated using a five-fold cross-validation within each fold, and the average calibration plot was evaluated. Calibration quality was quantified using the Brier score and the expected calibration error (ECE).

### RSV scoring

For each selected feature, the SHAP-dependent plots were used to determine the threshold values at which the probability of RSV infection increased. This was calculated from the integer decision point within a single decision tree. These thresholds were used to develop a simplified RSV scoring system for potential clinical applications.

### Additional analysis in children aged 1 year and older

Infants aged younger than 12 months were excluded because their pathophysiology of RSV, airway anatomy, immune maturation, and symptom presentation differ significantly from those of older children. To address this, we conducted an additional analysis restricted to patients aged ≥ 1 year (12 months or older). The methodology followed the steps described above with two key modifications. First, the analysis was limited to individuals aged one year or older. Second, due to the greater class imbalance, the CatBoostClassifier's class_weight parameter was enabled for analysis, in addition to the default setting. Synthetic Minority Over-sampling Technique (SMOTE) combined with simple imputation was also performed. The resulting scoring model was based on the top features selected using the mean SHAP values.

### Statistical analysis

Statistical analyses were performed using Python version 3.10.12. Statistical significance was defined as a two-sided p-value < 0.05.

## Results

### Data retrieval

The participants ranged in age from 1 to 78 months, and included 493 males and 275 females. Although males were more prevalent in the dataset, no statistically significant difference was observed in sex distribution between the RSV and non-RSV groups (*p* = 1.00). Significant differences were found between the two groups for all continuous variables, except heart rate (Table 1). No significant differences were observed in the frequency of upper respiratory symptoms such as cough (*p* = 0.70) and nasal discharge (p=0.26). In contrast, gastrointestinal symptoms, including vomiting and diarrhea, were significantly more frequent in the RSV group (Table 1). Regarding the chest auscultation findings, rhonchi were significantly more common in the RSV group, while stridor was significantly less common (Table 1). No significant differences in the presence of wheezing (*p*=0.13) or crackling (*p*=0.21) were observed. None of the continuous features demonstrated problematic collinearity (VIF < 5) (Additional file 2).

**Table 1:** Comparison of clinical and demographic characteristics between RSV-positive (case) and RSV-negative (control) groups

	Case (*n* = 135)	Control (*n* = 633)	*p*-value
Continuous variables			
Age (months), mean ± SD	14.3 ± 12.8	23.2 ± 15.7	< 0.05 ^¶^
Weight (kg), mean ± SD	9.11 ± 3.05	11.48 ± 3.66	< 0.05 ^¶^
Height (cm), mean ± SD	75.46 ± 13.68	82.90 ± 14.87	< 0.05 ^¶^
SpO_2_ (%), mean ± SD	93.82 ± 3.78	95.07 ± 4.34	< 0.05 ^¶^
Temperature (℃), mean ± SD	37.93 ± 0.87	37.71 ± 0.89	< 0.05 ^¶^
Respiratory rate (/min), mean ± SD	63.38 ± 14.13	55.93 ± 14.72	< 0.05 ^¶^
Heart rate (/min), mean ± SD	125.78 ± 22.34	127.21 ± 23.96	0.52
Categorical variables			
Gender, Male/Female (%)	87 (64)/48 (36)	406 (64)/227 (36)	1.00
Asthmatic history, Yes/No (%)	19 (14)/116 (86)	187 (30)/446 (70)	< 0.05 ^¶^
Vomiting, Yes/No (%)	82 (61)/53 (39)	306 (48)/327 (52)	< 0.05 ^¶^
Diarrhea, Yes/No (%)	45 (33)/90 (67)	109 (17)/523 (83)	< 0.05 ^¶^
Cough, Yes/No (%) ^§^	134 (99)/1 (1)	621 (98)/10 (2)	0.70
Rhinorrhea, Yes/No (%)	98 (73)/37 (27)	490 (77)/142 (23)	0.26
Stridor, Yes/No (%)	8 (6)/127 (94)	98 (15)/535 (85)	< 0.05 ^¶^
Rhonchi, Yes/No (%)	83 (61)/52 (39)	303 (48)/330 (52)	< 0.05 ^¶^
Crackles, Yes/No (%)	17 (13)/118 (87)	55 (9)/578 (91)	0.21
Wheezing, Yes/No (%)	97 (72)/38 (28)	409 (65)/224 (35)	0.13
Cyanosis, Yes/No (%)	11 (8)/124 (92)	56 (9)/577 (91)	0.93
Dehydration signs, Yes/No (%) ^§^	2 (1)/133 (99)	10 (2)/623 (98)	1.00
Nasal flaring, Yes/No (%)	88 (65)/47 (35)	465 (73)/168 (27)	0.07

^§^Fisher’s exact test was used because of the small sample size

^¶^Indicates statistically significant differences (*p* < 0.05)

### Machine learning and feature selection using mean SHAP values

**Fig. 1:**
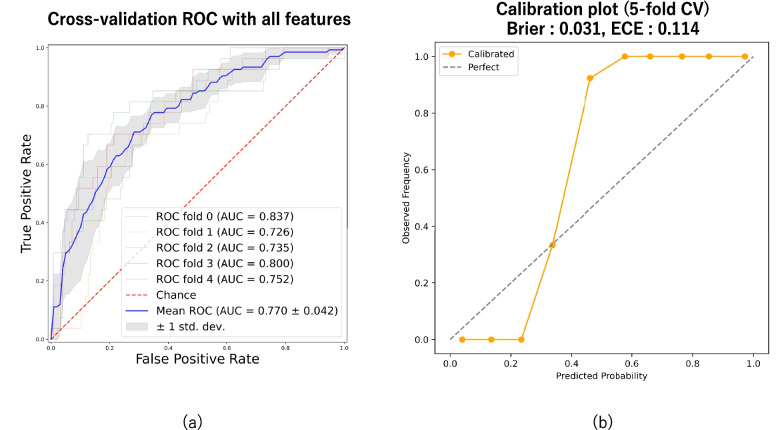
ROC curve and calibration plot for predicting RSV infection using ML models. **a** ROC curve based on all 20 features. **b** calibration plot showing the agreement between predicted probabilities and observed outcomes

**Fig. 2:**
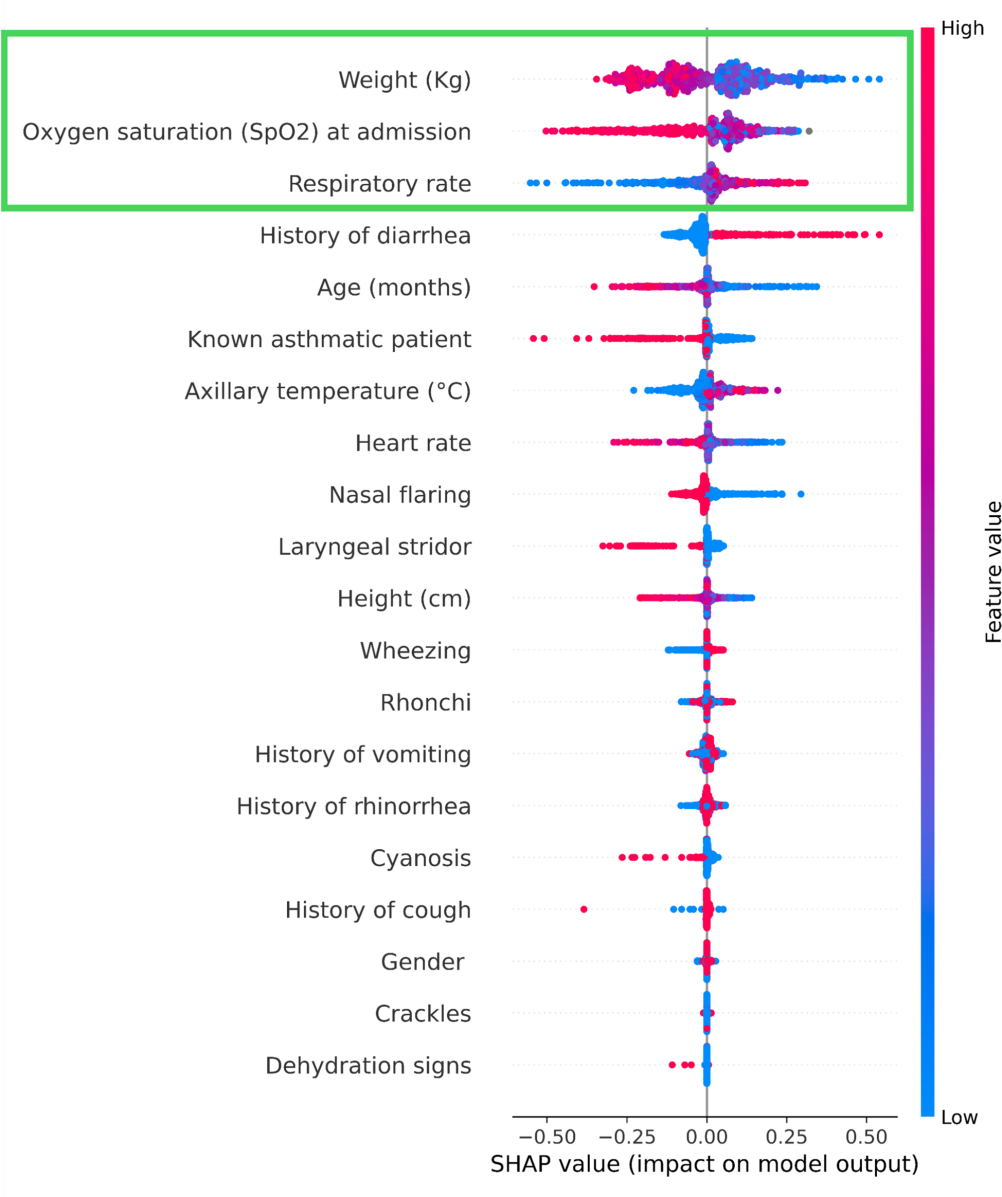
Feature importance based on mean SHAP values. Each point represents the SHAP value for a single prediction or feature. Red indicates higher feature values, and blue indicates lower values. The distributions of the red and blue points indicate the direction and magnitude of the impact of each feature on the model output. Features with a wider horizontal spread and higher SHAP values contributed significantly to the prediction of the model. The three features selected for further analysis are highlighted in green

**Fig. 3:**
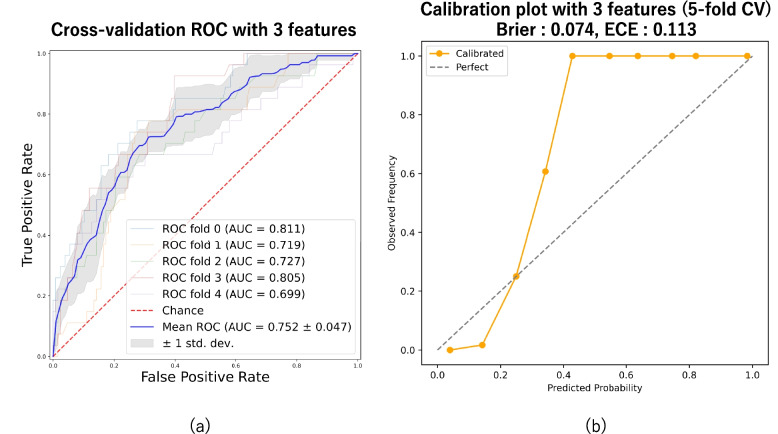
ROC curve and calibration plot using the three most important features. **a** ROC curve based on weight, SpO₂, and respiratory rate. **b** Calibration plot showing the agreement between predicted probabilities and observed outcomes

In the binary classification analysis using 20 clinical features, the AUC ranged from 0.726 to 0.837, with a mean AUC of 0.770, indicating moderate overall performance (Fig. 1a). Furthermore, when comparing RSV-only cases (*n* = 84) with the RSV-negative control group (*n* = 633), the AUC was 0.75 (Additional file 1). Feature importance was assessed using the mean SHAP values averaged across five-fold cross-validation. The three most important features were weight, SpO₂, and respiratory rate, in that order (Fig. 2). Reanalysis using only these three selected features yielded an AUC of 0.752, suggesting that a simplified model based on a small number of clinically relevant variables could still achieve moderate diagnostic performance (Fig. 3a).

### Calibration plot

That calibration is acceptable in the low-risk range but shows underestimation for higher predicted probabilities. In the quantitative evaluation, when all 20 features were used, the Brier score was 0.031, and the ECE was 0.114 (Fig. 1b). When only three features were used, the Brier score was 0.074 and the ECE was 0.113 (Fig. 3b). In both models, the calibration curves showed a consistent underestimation in the mid-to-high probability ranges. Notably, in the model using three variables, a predicted probability of approximately 50% corresponded to a subgroup in which all patients were RSV-positive, indicating substantial underconfidence.

### RSV scoring

**Fig. 4:**
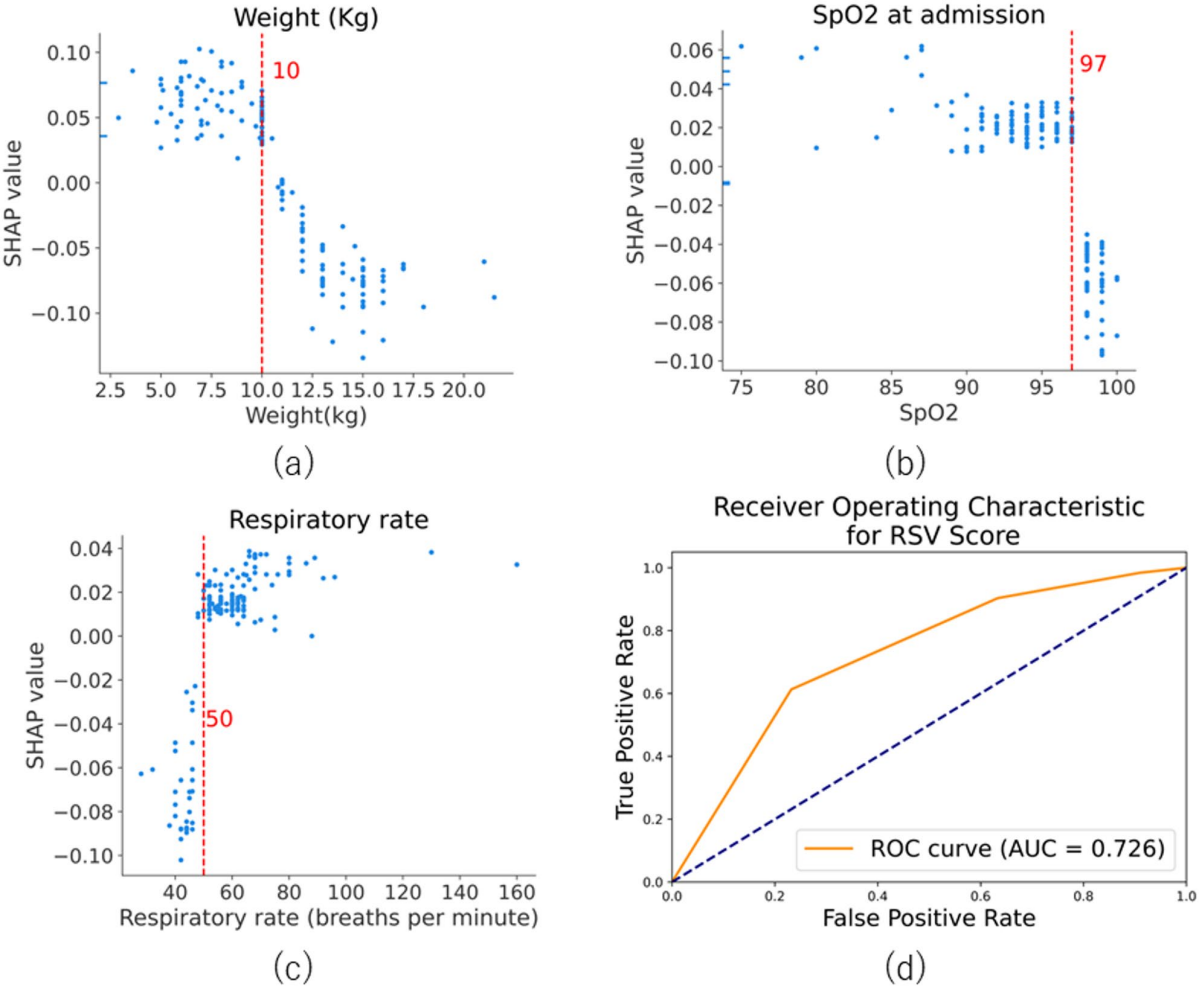
SHAP-derived thresholds and diagnostic performance of the RSV scoring system. **a**–**c** SHAP-dependence plots for weight, SpO₂, and respiratory rate, showing the threshold values associated with increased RSV probability. **d** ROC curve for the scoring system based on three selected features (AUC = 0.726)

Threshold values for the three continuous variables were determined using SHAP dependence plots: weight ≤ 10 kg, SpO₂ ≤ 97%, and respiratory rate ≥ 50 breaths per minute (Figs. 4a–4c). Each threshold was assigned one point, yielding a total possible score ranging from 0 to 3. When a score of two or higher was used as the cut-off, the sensitivity and specificity were 0.903 and 0.369, respectively (Table 2). In contrast, using a score of three as the threshold resulted in a sensitivity of 0.613 and a specificity of 0.768 (Table 2). The AUC for this scoring system was 0.726 (Fig. 4d), indicating moderate discriminatory ability.

**Table 2:** RSV scoring system based on three clinical features (maximum score: 3 points)

Weight	≤ 10 kg	1 point
Respiratory rate	≥ 50 breaths per minute	1 point
Oxygen saturation SpO_2_	≤ 97%	1 point

Using a cutoff score of ≥ 2 points: sensitivity = 0.903, specificity = 0.369. Using a cutoff score of three points, the sensitivity and specificity were 0.613 and 0.768, respectively.

### Additional analysis limited to patients aged one year or older

**Fig. 5:**
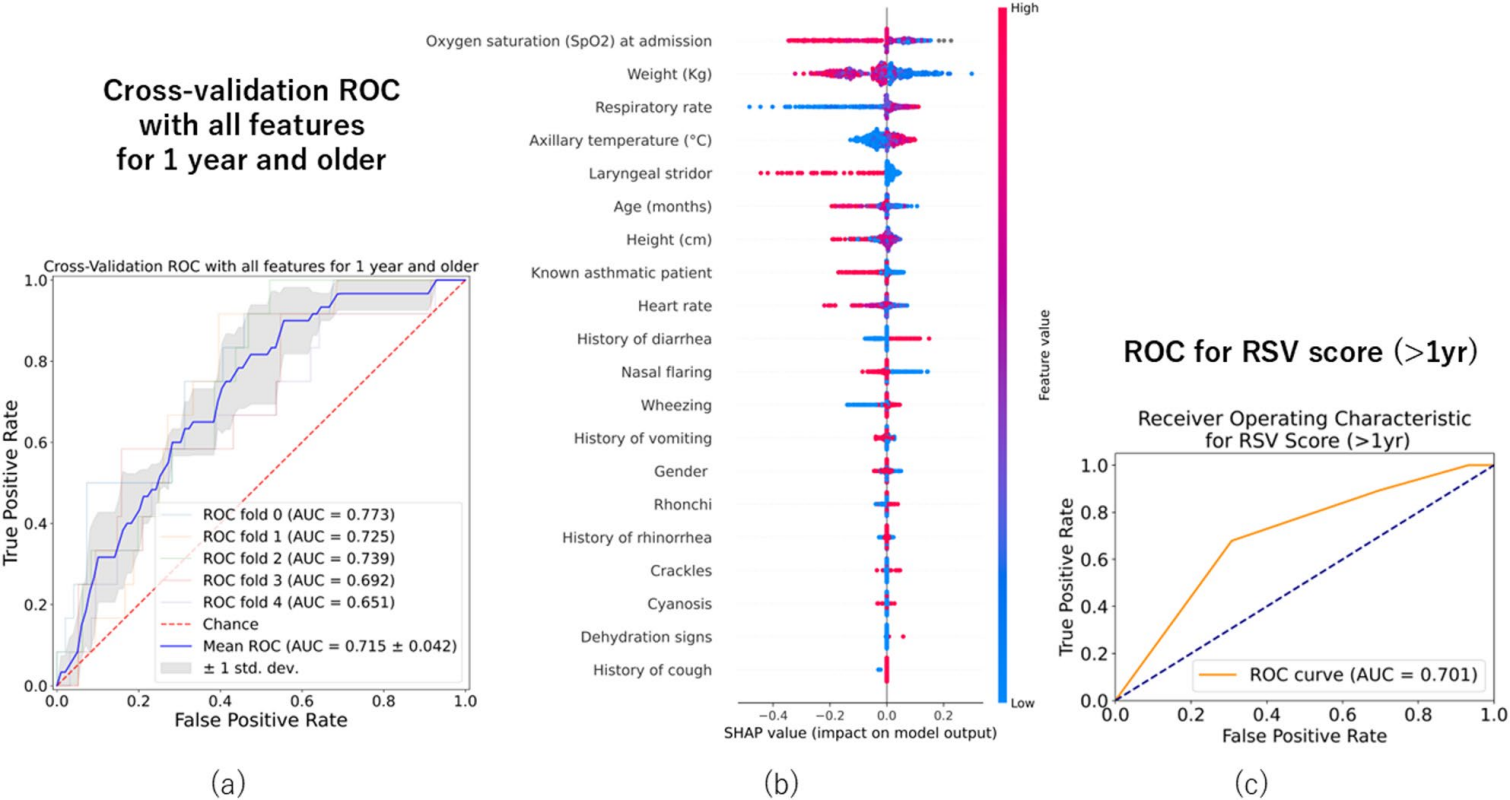
Additional analysis in children aged 1 year and older. **a** ROC curve for predicting RSV infection using all 20 features. **b** Feature importance ranked by average SHAP values. **c** ROC curve for the RSV scoring system based on four features: weight, SpO_2_, respiratory rate, and stridor (AUC = 0.701)

When the analysis was restricted to patients aged one year or older, the number of RSV-positive cases was reduced to 60, while 478 patients served as RSV-negative controls. Using all 20 explanatory variables, the mean AUC from the five-fold cross-validation was 0.715, indicating moderate predictive performance (Fig. 5a). The measures for handling imbalanced data were unsuitable for the class_weight parameter and SMOTE (Additional file 3). The top three features based on the mean SHAP values were SpO₂, weight, and respiratory rate, which were consistent with the findings from the full cohort (Fig. 5b). Threshold values derived from SHAP dependence plots were SpO₂ ≤ 97%, weight ≤ 14 kg, and respiratory rate ≥ 45 breaths per minute. An RSV scoring system was developed by assigning one point to each threshold variable. The presence or absence of stridor was included as an additional binary variable based on clinical relevance, resulting in a possible total score ranging from 0 to 4 (Table 3). A score of ≥ 3 yielded a sensitivity of 0.893 and a specificity of 0.342. A score of four resulted in a sensitivity of 0.679, a specificity of 0.693, and an AUC of 0.701 (Fig. 5c), indicating moderate discriminatory ability.

**Table 3:** RSV scoring system for children aged one year and older (maximum score: 4 points)

Weight	≤ 14 kg	1 point
Respiratory rate	≥ 45 breaths per minute	1 point
SpO_2_	≤ 97%	1 point
Stridor	Absent	1 point

Score ≥ 3: sensitivity = 0.893, specificity = 0.342. When the score was 4 out of 4, the sensitivity and specificity were 0.679 and 0.693, respectively.

## Discussion

In this study, the most important clinical features for predicting pediatric RSV infection were weight, SpO₂, and respiratory rate. The average AUC across the five cross-validation folds was 0.770, indicating moderate predictive performance. A simplified scoring system based on threshold values for weight (≤ 10 kg), respiratory rate (≥ 50 breaths/min), and SpO₂ (≤ 97%) achieved an AUC of 0.726. Additionally, a separate scoring model was developed for children aged 1 year and older. This adapted model, which incorporated the same three core features and the presence or absence of stridor, also demonstrated moderate performance (AUC = 0.715), suggesting its potential utility in clinical settings.

ML applications in the diagnosis of infectious diseases have expanded rapidly in recent years. Several studies have explored ML approaches for RSV detection and severity assessment [14–19]. For example, Tso et al. developed an XGBoost model using electronic health record data and reported high diagnostic performance (AUC = 0.919) [14]. Kawamoto et al. used self-reported symptom data from infants to construct a model that identified cases in which antigen testing could potentially be avoided [15]. De Iaco et al. proposed a LightGBM model based on clinical symptoms and laboratory findings, demonstrating its potential for low-cost diagnostic support that does not require PCR testing [16]. Soriano-Arandes et al. applied a random forest model to outpatient symptom data and demonstrated its feasibility in primary care settings [17]. Sun et al. introduced an online transfer learning model that was dynamically adapted to real-time clinical data [18], and Liu et al. employed gene expression data to build a neural network for predicting severe RSV pneumonia [19]. Collectively, these studies illustrate the versatility of ML in supporting RSV-related clinical decision-making from symptom-based triage to molecular-level prediction. However, despite the promise of ML-based systems, their implementation often requires a specialized infrastructure, large annotated datasets, and substantial computational resources. Therefore, these systems may not be feasible in all clinical environments, particularly in resource-limited settings. In this context, our simple scoring system, based on objective continuous variables with interpretable thresholds, may serve as a more practical tool for frontline clinicians.

Accurate diagnosis and severity assessment of RSV infections in children remain critical challenges in clinical pediatrics. Several scoring systems have been developed to aid clinicians in evaluating RSV infection [20–24], each with distinct strengths depending on the clinical context and patient population. For example, the RSV-CLASS is a concise and clinically intuitive tool that includes four features: cough, tachypnea, rales, and wheezing. It demonstrated strong performance, with an AUC of 0.90 in the development cohort and 0.87 in external validation [20]. Its simplicity renders it suitable for use in outpatient clinics. However, a major limitation is that it was developed during the RSV epidemic season (i.e., winter months), which may limit its generalizability in non-seasonal or tropical settings. For a more detailed severity assessment, the Global Respiratory Severity Score (GRSS) has demonstrated excellent performance (AUC = 0.961) and correlates well with hospitalization needs in infants aged < 10 months of age [21]. The GRSS incorporates nine features derived from factor analysis. Notably, both SpO₂ and respiratory rate, features selected in our model, were also included in the GRSS, underscoring their clinical importance. However, the GRSS was developed using a relatively small cohort of young infants, and its complexity limits its applicability in routine clinical practice. It is primarily used in research rather than in bedside settings. Our simple scoring system is less accurate than complex ML models but offers immediate usability and greater versatility in clinical practice. Specifically, by focusing on measurable, objective vital signs across diverse settings, including children aged one year and older, this scoring system can complement existing tools whose validity is largely restricted to infants in specific epidemiological contexts.

The primary purpose of this score as a triage tool is to prioritize diagnostic testing and isolation for children suspected of having severe lower respiratory tract infection, and it does not predict clinical deterioration itself. This scoring system is useful for estimating the number of patients with RSV infection requiring hospitalization and holds potential value as a clinical decision-support tool. Particularly in settings where it is challenging to perform rapid antigen or PCR testing for all patients, prioritizing testing for patients with high scores may facilitate efficient allocation of testing resources and earlier isolation. Furthermore, in outpatient or emergency settings, the score may facilitate rapid decision-making on oxygen administration or hospitalization criteria, enabling implementation as a triage support tool. The three primary predictors identified by SHAP, including weight, SpO₂, and respiratory rate, all align with the pathophysiology of RSV [1–7]. Specifically, increased respiratory effort and impaired oxygenation due to airway inflammation are involved, particularly prominent in younger and lower birth weight infants. Interestingly, the top-ranked clinical predictors, including weight, SpO₂, and respiratory rate, remained stable in both RSV-only and coinfection subgroups. This indicates that the scoring system primarily reflects the host physiological response to lower respiratory tract inflammation, rather than pathogen-specific mechanisms.

A major strength of this study was the reuse of a publicly available dataset to derive new insights using interpretable ML techniques. By focusing on objective continuous variables, we developed a model that performed reasonably well without relying on subjective clinical assessments. Moreover, the derived scoring system is straightforward and can be readily implemented in clinical workflows, offering practical value in settings in which diagnostic testing is limited or delayed.

This study has several limitations. First, that the analysis was based on data from a single institution in Morocco may have limited the generalizability of the findings. To be more specific, potential regional differences, including ethnicity, nutritional background, climate-dependent RSV seasonality, healthcare accessibility, and hospitalization thresholds, may limit the generalizability of the scoring system. Second, the study population included only children with severe pneumonia, as defined by the WHO, potentially narrowing the clinical spectrum of RSV presentations captured in the dataset. Third, although our models demonstrated good calibration in the low-risk ranges, underestimation of the actual risk was observed at higher predicted probabilities, particularly in the simplified three-variable model. This underconfidence highlights the need for caution when using predicted probabilities for direct clinical decision-making and underscores the importance of external validation and recalibration. Finally, although robust features were identified through cross validation, external validation using independent datasets is essential to confirm the reproducibility and clinical utility of the proposed scoring system. The model is not yet suitable for estimating precise risk probabilities, but remains clinically valuable for supporting triage, testing prioritization, and early decision-making.

To further improve this model, it is essential to evaluate its clinical utility by introducing blood test parameters, expanding the sample size, and broadening the target age range. In the future, we plan to perform external validation through a prospective, multicenter collaborative study to verify the model's validity and implementation feasibility.

## Conclusions

Although many viruses can cause respiratory infections in children, this study indicates that testing and treatment of RSV are particularly important when changes in body weight, respiratory rate, and SpO₂ are observed. Clinical assessment based on these objective indicators may support the timely and accurate diagnosis of RSV and contribute to more appropriate and effective patient management.

## Supplementary Information

Additional file 1. Analysis of RSV-only cases (*n* = 84), excluding coinfections, versus non-RSV controls (*n* = 633).

Additional file 2. The variance inflation factor for continuous variables

Additional file 3. ROC plot for patients aged one year and older as the: (a) default setting, (b) “class_weight” parameter, and (c) simple imputation and SMOTE

## Data Availability

A publicly available dataset on pediatric respiratory infections was obtained from Mendeley Data: Pediatric Respiratory Infections: Epidemiological and Etiological Data from a Cohort of 801 Moroccan Children (https://data.mendeley.com/datasets/2d9dvnycjw/1). The dataset used in this study is publicly available under the Creative Commons Attribution 4.0 International License (CC BY 4.0), which permits unrestricted reuse, analysis, and modification with proper attribution. The code used in this analysis is available on GitHub (https://github.com/YoshifumiMiyagi/RSV_analysis).

## Abbreviations

RSV: Respiratory syncytial virus
ML: Machine learning
SpO_2_: Oxygen saturation
SHAP: SHapley Additive exPlanations
ROC: Receiver operating characteristic
AUC: Area under the receiver operating characteristic curve
WHO: World Health Organization
ECE: Expected calibration error
PCR: Polymerase chain reaction
MLPA: Multiplex ligation-dependent probe amplification
VIF: Variance inflation factor
