# Supplementary figures and images for: Identification of key clinical features for pediatric respiratory syncytial virus infection using machine learning
Source: BMC Pediatr. 2026 Feb 27;26:266. doi: 10.1186/s12887-026-06659-z (PMC13049749; doi:10.1186/s12887-026-06659-z)

## Slide 1
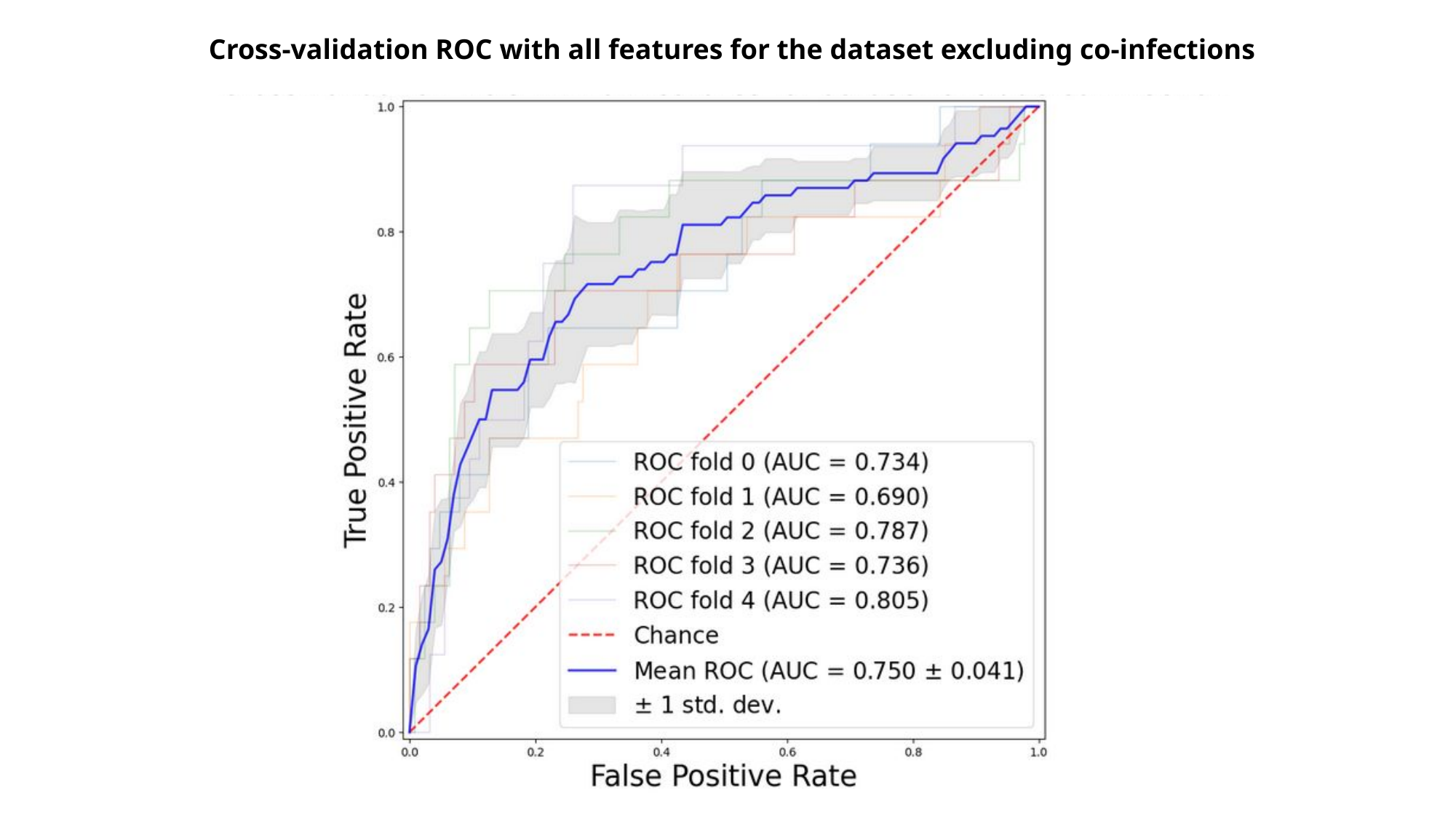

Cross-validation ROC with all features for the dataset excluding co-infections

Supplement: Supplementary file 1 — Additional file 1. Analysis of RSV-only cases (n = 84), excluding coinfections, versus non-RSV controls (n = 633). [file 12887_2026_6659_MOESM1_ESM.pptx]

## Slide 1
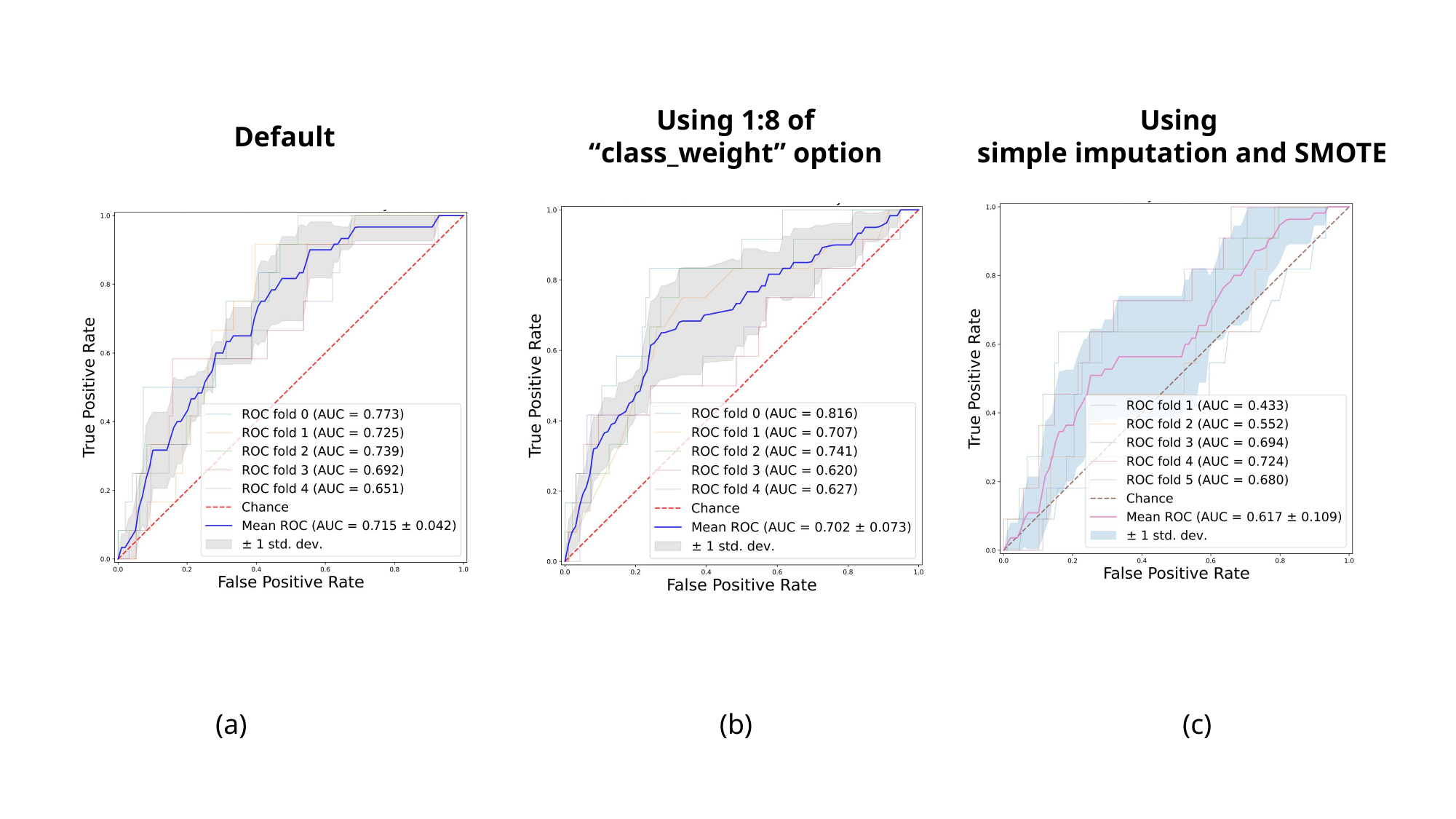

Using 1:8 of
“class_weight” option
Using
simple imputation and SMOTE
Default
(a)
(b)
(c)

Supplement: Supplementary file 3 — Additional file 3. ROC plot for patients aged one year and older as the: (a) default setting, (b) “class_weight” parameter, and (c) simple imputation and SMOTE [file 12887_2026_6659_MOESM3_ESM.pptx]
