# Supplementary material for: Identification of key clinical features for pediatric respiratory syncytial virus infection using machine learning
Source: BMC Pediatr. 2026 Feb 27;26:266. doi: 10.1186/s12887-026-06659-z (PMC13049749; doi:10.1186/s12887-026-06659-z)
Supplement: Supplementary file 2 — Additional file 2. The variance inflation factor for continuous variables [file 12887_2026_6659_MOESM2_ESM.pptx]

## Slide 1
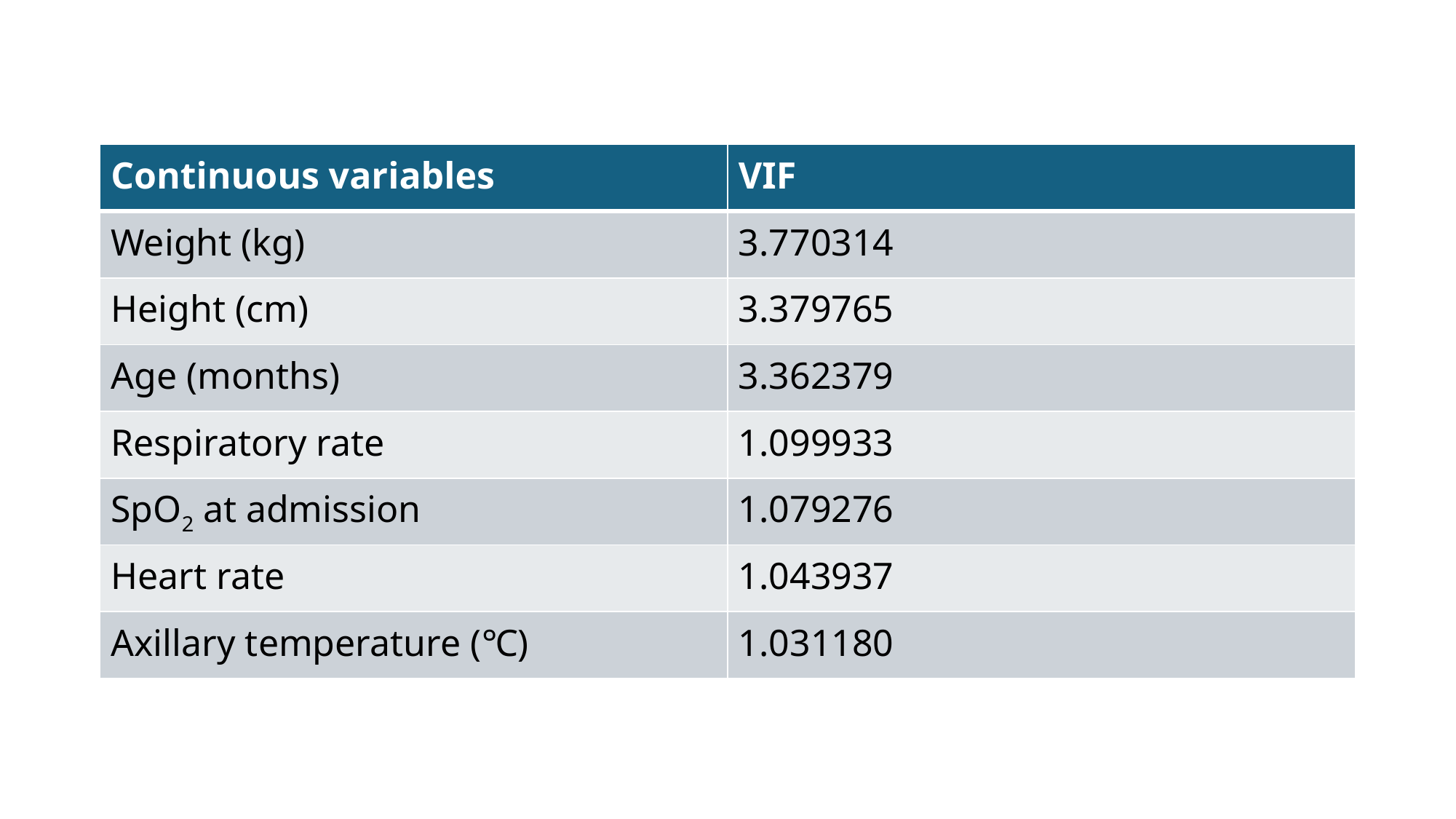

| Continuous variables | VIF |
| --- | --- |
| Weight (kg) | 3.770314 |
| Height (cm) | 3.379765 |
| Age (months) | 3.362379 |
| Respiratory rate | 1.099933 |
| SpO2 at admission | 1.079276 |
| Heart rate | 1.043937 |
| Axillary temperature (℃) | 1.031180 |
